# Analysis of the clinical characteristics of adult patients with hangman's fractures: A retrospective study based on multicenter clinical data

**DOI:** 10.3389/fsurg.2023.949987

**Published:** 2023-04-04

**Authors:** Guangzhou Li, Qing Wang

**Affiliations:** The Affiliated Hospital of Southwest Medical University, Luzhou, China

**Keywords:** hangman's fracture, clinical characteristics, retrospective study, age group, treatment

## Abstract

**Background:**

There are few reports on the clinical characteristics of adult patients with hangman's fractures.

**Methods:**

The clinical data of adult patients were collected from the hangman's fracture database of 7 medical centers. Data on patients who met the inclusion and exclusion criteria were retrospectively analyzed. Data, including gender, age, mechanism of injury, fracture classification, and treatment, were statistically analyzed.

**Results:**

A total of 216 eligible patients (160 males and 56 females, with a mean age of 49.7 years) were selected. There was no statistically significant difference in gender distribution of different age groups. The male-to-female ratio was similar in the young group (18–44 years) and the middle-aged group (45–64 years) (both about 3:1) but decreased in the elderly group (65 years and above) (about 2:1). Overall, high-energy injury was the main mechanism of injury. There was a statistically significant difference in the percentage of patients with high-energy injury in various age groups (the highest in the young group, and the lowest in the elderly group). Overall, unstable fracture was the main fracture type, with a higher proportion in the young and elderly groups than that in the middle-aged group, but there was no statistically significant difference. From the perspective of treatment options, the percentage of patients receiving surgery was higher in the young and elderly groups than that in the middle-aged group.

**Conclusion:**

Hangman's fracture is predominant in males of all age groups, with high-energy injury as the main mechanism of injury. Unstable fracture is common fracture type. The percentage of patients receiving surgery in the young and elderly groups is higher than that in the middle-aged group, which may be correlated with the high incidence of unstable fracture and the life characteristics of the patients in the young and elderly age groups.

## Introduction

Hangman's fracture, also known as “axis ring fracture” or traumatic spondylolisthesis of axis, is a common injury of the upper cervical spine (1–6). In the literature, it has been reported that hangman's fractures account for 4%–7% of spinal fractures and about 1/5 of cervical vertebral fractures (1, 3, 6).

Some of the hangman's fractures are unstable fractures. If insufficient attention is paid, inappropriate diagnosis and treatment will not only affect the rehabilitation of the patients but also increase their economic burden. Simultaneously, with the increasing aging of the population and the dramatic changes in the current social lifestyle, the composition of patient population and injury patterns may also change (2, 6–8). Therefore, it is of significance to analyze the clinical characteristics of different age groups adult patients with hangman's fractures, which may help provide comprehensive data to medical staff in the emergency department and orthopedics center (or spine center).

In the last 20 years, there has been no English literature on the analysis of the clinical characteristics of patients with hangman's fractures based on a large sample size (4, 8–10). In this study, the clinical characteristics of adult patients with hangman's fractures were analyzed with data, including gender, age, mechanism of injury, fracture classification, and treatment, which were collected from a hangman's fracture database jointly established by multiple medical centers from across China.

## Materials and methods

### Inclusion and exclusion criteria

Inclusion criteria included the following: (1) fresh (fractures within 10 days of the injury) or old hangman's fractures (11); (2) receiving conservative or surgical treatment; (3) having complete data of gender, age, mechanism of injury, fracture classification, and treatment; (4) having complete results of x-ray and 3D CT of the cervical spine before treatment. Exclusion criteria included the following: (1) age less than 18 years; (2) pathological fracture; (3) combined with cervical deformity, infection, and congenital dysplasia.

This research has been approved by the IRB of the authors' affiliated institutions. Totally, 241 patients who sustained Hangman fractures were reviewed, and 25 patients were excluded, including 17 patients with incomplete medical records or images, 3 with age of less than 18 years, 3 with congenital deformity, and 2 with congenital dysplasia. Finally, our series included 216 patients.

### General information

Relevant information about patients meeting the inclusion and exclusion criteria was searched in the hangman's fracture database (the data from 7 medical centers across China) from October 2008 to December 2020. Data, including gender, age, mechanism of injury, fracture classification, and treatment of the patients, were statistically analyzed.

### Grouping

According to the age, the patients were divided into the young group (18–44 years), the middle-aged group (45–64 years), and the elderly group (65 years and above) (12–15).

### Observational indicators

#### Fracture classification

Patients' fractures were classified according to Levine–Edwards classification into stable fractures (Levine–Edwards type I) and unstable fractures (Levine–Edwards types II, IIa, and III) (4, 5).

#### Mechanisms of injury

The mechanism of injuries registered in the database was classified into four types: motor vehicle accident, falling from a height, falling on a flat surface (or falling over), and others (such as strike by heavy objects or unknown cause and mechanism of the injury). In this study, injury caused by motor vehicle accident or falling from a height or strike by heavy objects was classified as high-energy injury, falling on a flat surface (or falling over) injury as low-energy injury, and unknown cause and mechanism of the injury as unknown (14, 15).

#### Conditions of neurological injury

According to the American Spinal Injury Association (ASIA) score (16), patients with hangman's fracture combined with spinal cord injury registered in the database were graded as A, B, C, D, and E.

#### Treatments and outcomes

Treatments were classified into anterior cervical surgery, posterior cervical surgery, anterior–posterior approach surgery, and conservative treatment (17–20). For patients whose 1 year or more of follow-up data could be obtained, Odom's grading system (excellent, good, fair, or poor) was used to assess the clinical outcomes after treatment (17).

### Statistical analysis

Data were analyzed using SPSS 22.0 statistical software (SPSS, USA). Measurement data were expressed as mean ± standard deviation and compared between groups using independent sample *t*-test. An independent sample nonparametric test will be used if the variance is heterogeneous. Enumeration data, including gender, fracture type, injury mechanism, and treatment, was analyzed using the chi-squared or Fisher exact test. The statistical significance level was set at *α *= 0.05 and *P *< 0.05.

## Results

### Demographic characteristics

A total of 216 eligible patients (160 males and 56 females) were selected, with a mean age of 49.6 ± 15.6 years (49.4 ± 15.1 and 50.0 ± 16.8 in the male and female groups, respectively) and a range from 21 to 91 years. There was no statistical difference between the two groups (*T* = 0.236, *P *= 0.814).

According to patients' age, 85, 91, and 40 patients were included in the young group, the middle-aged group, and the elderly group, respectively. Although the male patients outnumbered the female patients overall and in each age group, the percentage of the female patients in the elderly group increased (Table 1). The male-to-female ratio was about 3:1 in the young and middle-aged groups but about 2.1:1 in the elderly group. There was no significant difference in gender distribution among the three age groups (*P *> 0.05).

**Table 1:** Comparison of gender and fracture types in different age groups.

Groups	Case number	Gender		Fracture types	
		Male	Female	Unstable fracture	Stable fracture
The young group	85	64	21	49	36
The middle-aged group	91	69	22	42	49
The elderly group	40	27	13	22	18
*χ*^2^ value	–	1.111		2.469	
*P* value	–	.574		.291	

### Type of fracture

Overall, as per the Levine–Edwards classification, there were 113 patients with unstable fractures (84, 20, and 9 for types II, IIa, and III, respectively) and 103 patients with stable fractures, with a slight majority of unstable fractures (52.3%, 113/216). The percentage of unstable fractures in the young and elderly groups (57.6% and 55% respectively) accounted for more than half of all fractures, which was higher than that in the middle-aged group (46.2%). However, there was no statistically significant difference (*P *> 0.05, Table 1).

### Injury mechanism

There was a statistically significant difference in the distribution of injury mechanism among the three groups (*P* < 0.001, Table 2). Further, high-energy injury was the primary mechanism in the three age groups (96.5%, 78%, and 52.5% of high-energy injury in the young, middle-aged, and elderly group, respectively). The percentage of high-energy injuries in the young and middle-aged groups was significantly higher than that in the elderly group (for both, *P* < 0.001). The percentage of high-energy injuries in the young group was significantly higher than that in the middle-aged group (*χ*^2 ^= 13.167, *P* < 0.001).

**Table 2:** Comparison of injury mechanism and neurological injury in different age groups.

Groups	Case number	Injury mechanism			Neurological injury	
		High-energy injury	Low-energy injury	Unknown	Yes	No
The young group	85	82	1	2	19	66
The middle-aged group	91	71	15	5	14	77
The elderly group	40	21	19	0	2	38
*χ*^2^ value	–	45.612			6.110	
*P* value	–	<.001			.047	

### Neurological injury

Overall, 35 patients had neurological injuries caused by hangman's fracture, with an incidence of 16.2% (35/216). As per the ASIA scale, there were 1 case of grade B, 4 cases of grade C, and 30 cases of grade D. In the young group, 19 patients had neurological injuries (22.4%, 19/85), including 15 cases of grade D and 4 cases of grade C. In the middle-aged group, 14 patients had neurological injuries (15.4%, 14/91), including 13 cases of grade D and 1 case of grade B. In the elderly group, 2 patients had neurological injury of grade D (5%, 2/40) (Table 2). The incidence of neurological injury was the highest in the young group and decreased gradually with the increase in age (22.4%, 15.4%, and 5%, respectively). There was a significant difference among the three groups (*χ*^2^ = 6.110, *P *= 0.047). Paired comparison showed that the incidence of neurological injury in the young group was significantly higher than that in the elderly group (*χ*^2^ = 5.860, *P *= 0.015). There was no significant difference in the incidence of neurological injury between the young and middle-aged groups (*χ*^2^ = 1.401, *P *= 0.237) and between the middle-aged and elderly groups (Fisher's exact test, *P *= 0.146).

### Treatments and outcomes

There were statistically significant differences in treatment options (surgery or conservative treatment) among the three groups (*χ*^2^ = 10.045 and *P *= 0.007), with a higher percentage of patients treated with internal fixation in the young and elderly groups (65.9% and 65%) than that in the middle-aged group (44%) (*χ*^2^ = 8.522 and 4.922; *P *= 0.004 and 0.027, respectively, Table 3). Surgical treatments with anterior cervical surgery, posterior cervical surgery, and anterior–posterior approach surgery, were used in 12, 41, and 3 patients in the young group. Surgical treatments with aforementioned approaches were used in 9, 29, and 2 patients in the middle-aged group, and 3, 22, and 0 patients in the elderly group, respectively (Table 3) (17–20). For unstable hangman's fractures, posterior cervical surgery was the most common used approach in all different age groups, and posterior C2-3 fixation was preferred option (5, 18, 19).

**Table 3:** Comparison of treatments in different age groups.

Groups	Case number	Treatments			
		Anterior surgery	Posterior surgery	Anterior-posterior approach surgery	Conservative treatment
The young group	85	12	41	3	29
The middle-aged group	91	9	29	2	51
The elderly group	40	3	22	0	15
*χ*^2^ value	–	10.045			
*P* value	–	.007			

Data of 134 patients with 1 year or more of follow-up was obtained, and 113 (84.3%, 113/134) patients rated their level of satisfaction as excellent or good, according to Odom's criteria (17).

## Discussion

In this study, we divided the adult patients into different age groups, mainly because of differences in health status, bone condition, lifestyle, recreational interests, job position, and other factors among different age groups (14, 15). Previous studies showed that patients with hangman's fracture are mainly young and middle-aged adults, with a relatively lower percentage of elderly patients (1–5). This study also showed a similar result that 81.5% of the patients with hangman's fractures were aged 18–64 years, and 18.5% were elderly patients. Although the percentage of the elderly patients is lower than that of the middle-aged patients, the incidence of unstable fractures in the elderly group is 55%, and conservative treatments, such as traction and prolonged immobility, are more probably associated with bed-related complications. Therefore, more attention should be paid to elderly patients with hangman's fractures.

Although most of the patients with hangman's fractures are males, with a higher percentage of males in all age groups, the percentage of females in the elderly group is increasing. This may be related to the fact that males in all age groups are engaged in more physical labor, more active, and more likely to be injured than females, while the increased incidence of hangman's fracture in the elderly females is associated with postmenopausal osteoporosis (14, 15).

In this study, as per the Levine–Edwards classification, hangman's fractures were divided into unstable and stable fractures. The study results showed a slightly higher incidence of unstable fractures than stable fractures, where the patients with unstable fractures accounted for over 50% in the young group and the elderly group (57.6% and 55%, respectively), but less than 50% (46.2%) in the middle-aged group. In the aspect of injury mechanism, this study showed that fractures in more than half of the patients of all age groups were caused by high-energy injury, with the highest percentage in the young group, followed by the middle-aged group and the elderly group. Paired comparison showed that there were statistically significant differences between any two groups. In other words, this study showed that nearly half of hangman's fractures of the elderly group were caused by low-energy injuries, and the distribution of low-energy injuries was higher than that of other two groups. There were few studies investigating the clinical characteristics of hangman's fractures in the elderly people, and two studies investigating the clinical characteristics of the elderly patients with odontoid fractures showed similar findings as our results (14, 15). The higher percentage of unstable fractures might be related to the higher percentage of high-energy injuries in the young group and factors such as osteoporosis and ligament laxity in the elderly group (5, 14, 15).

The study results showed that the incidence of neurological injury caused by hangman's fracture was 16.2%, which was basically consistent with the results of previous studies (1, 3, 5, 21–23); 35 patients with neurological injury (1 case of grade B, 4 cases of grade C, and 30 cases of grade D) all had incomplete neurological injuries, and most of them had mild neurological impairment. The incidence of neurological injury was the highest in the young group, followed by the middle-aged group, and the lowest in the elderly group. The incidence of neurological injury in the young group was significantly higher than that in the elderly group. The reason for the highest incidence of neurological injury in the young age group might be related to the higher percentage of high-energy injuries in this group, whereas the percentage of low-energy injuries in the elderly group was the highest. This study suggests that the neurological injury caused by hangman's fracture is closely correlated with the energy of injury, just like that of thoracolumbar burst fractures (24).

This study showed that more than half (56%, 121/216) of hangman's fractures were surgically treated, roughly equivalent to the percentage of unstable fractures in this study population (52.3%, 113/216). Generally, the majority of unstable hangman's fractures were treated by surgery, while most of the stable fractures were treated conservatively, and partial stable fractures received a surgery, which is consistent with the opinions reported in recent years (1–3, 5, 25, 26).

Specifically, the percentage of patients treated with internal fixation was higher in the young and elderly groups (65.9% and 65%) than that in the middle-aged group (44%). In the young group, the possible reasons for adopting internal fixation were as follows: the patients in the young group could not endure long-time traction or immobilization as they were more active and had a high demand for mobility; in the young group, the incidence of neurological injury was the highest; compared with the middle-aged group, the young group had a higher incidence of unstable fractures. The reason why the percentage of patients receiving internal fixation was higher in the elderly group than that in the middle-aged group might be due to the following: compared with the middle-aged group, the incidence of unstable hangman's fracture was higher in the elderly group (2, 4, 5, 19, 20); compared with the surgical treatment, the incidence of complications caused by traction and prolonged immobilization in the conservative treatment might be higher in the elderly group (14, 15). The reasons for the higher percentage of conservative treatment in the middle-aged group compared with the other two groups might be that the percentage of patients with stable hangman's fracture was higher in the middle-aged group, and most of the patients in this group were the main economic support of the family (4, 5, 17–19, 26).

Our experiences for treatment of hangman's fractures in adult patients are listed as follows: first, Levine–Edwards classification is primarily used, and unstable fractures (Levine–Edwards types II, IIa, and III) are better to be treated with surgical fixation, and posterior surgery is preferred (4, 5, 18, 19); second, some supplementary classifications for hangman's fractures could be applied to analyze the anatomical features of such fractures, which are of importance when posterior C2-3 pedicle screw technique is used (25–27); third, Levine–Edwards type I fractures should be also evaluated cautiously, and if these fractures were found with neurological deficit and/or instability, surgical fixation should be performed (26, 27); last, for hangman's fractures with superior facet joints injuries obvious displacement, posterior fixation at C2-3 level and C1-2 temporary fixation might be a proper surgical technique, avoiding sacrificing the movement of C1-2 (1, 25).

This study was conducted based on the hangman's fracture data from orthopedic or spine surgery departments in multiple medical centers. Although it can reflect the clinical characteristics of adult patients with hangman's fractures to some extent, it is a retrospective study with a limited sample size. Thus, a further prospective study with a larger sample size is needed.

## Conclusions

In conclusion, the percentage of males with hangman's fracture was higher overall and in all age groups, with high-energy injury as the main mechanism of injury. Unstable fracture was the most common fracture type. The percentage of patients treated with internal fixation was higher in the young and elderly groups than that in the middle-aged group due to the high incidence of unstable fracture and the characteristics of the age groups.

## Data Availability

The original contributions presented in the study are included in the article/Supplementary Material, further inquiries can be directed to the corresponding author.
